# Supplementary material for: Characterizing the Randot Preschool stereotest: Testability, norms, reliability, specificity and sensitivity in children aged 2-11 years
Source: PLoS One. 2019 Nov 7;14(11):e0224402. doi: 10.1371/journal.pone.0224402 (PMC6837395; doi:10.1371/journal.pone.0224402)
Supplement: S1 File — (DOCX) [file pone.0224402.s001.docx]

# Supporting Information

## 1. Normative data with more stringent limits on visual acuity

Excluding children in whom visual acuity was not measured to threshold, or where it was worse than 0.1 logMAR in either eye, or where the visual acuity difference exceeded 0.1 logMAR, we obtain the normative values shown in Table 1. These are lower than with the cut-offs of 0.2 logMAR used in the main paper, but still a little higher than other studies have reported. For example, Birch et al (2008) found a mean of 60 arcsec for 6-year-olds and 40 arcsec for 7-8 and 9-10-year-olds, while our values are 73 and 53 respectively.

| Age-group | Number tested | % scoring nil | Mean, SD computed on those who could complete at least 800 arcsec | | | | Percentiles computed on all tested | | | | |
| --- | --- | --- | --- | --- | --- | --- | --- | --- | --- | --- | --- |
|  |  |  | Threshold in arcsec | | Log-threshold in log_10_ arcsec | | Percentile in arcsec | | | | |
|  |  |  | Mean | SD | Mean | SD | 25% | 50% | 75% | 90% | 95% |
| 6 & 7yo | 158 | 0.0 | 73.4 | 99.3 | 1.74 | 0.257 | 40 | 40 | 60 | 100 | 200 |
| 10 & 11yo | 151 | 0.0 | 52.5 | 65.7 | 1.66 | 0.167 | 40 | 40 | 40 | 60 | 100 |
| All | 510 | 3.3 | 133. | 193. | 1.89 | 0.390 | 40 | 60 | 100 | 400 | 800 |

Table 1. Randot Preschool stereo thresholds by age-group, for children in the older age-groups who had visual acuity better than normal vision who were judged as being able to understand and cooperate with the test. Mean and SDs are reported for those children who could complete at least the 800 arcsec test level. Percentiles (type 1 quantile from Hyndman and Fan (1996)) are for all children, including those who scored “nil”. Stereothresholds in the normal range for each age group are marked in green (up to the 75% percentile). “Nil” means unable to perform 800 arcsec plate of Randot Preschool despite passing non-stereo pre-test.

## 2. Sensitivity and specificity for limited data set where full data were available

We also examined the data after excluding children for whom parental questionnaires were not available. This left 480 children aged 2 to 11 years. The results were similar. The specificity was similarly high and the sensitivity was slightly worse.


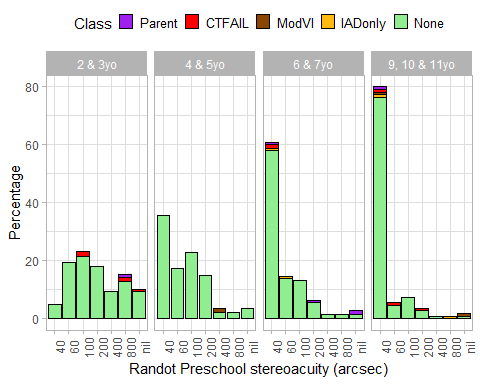


Figure 1. As previous except excluding children for whom visual acuity or parental questionnaire were not available.

Table 2. Specificity and sensitivity using restricted data=set (excluding children where visual acuity or parental questionnaire was not available)

|  | N | TP | FP | TN | FN | Sensitivity | Specificity | PPV | NPV |
| --- | --- | --- | --- | --- | --- | --- | --- | --- | --- |
| 2 & 3yo | 139 | 1 | 13 | 120 | 5 | 17 | 90 | 7 | 96 |
| 4 & 5yo | 87 | 0 | 3 | 83 | 1 | 0 | 97 | 0 | 99 |
| 6 & 7yo | 145 | 2 | 2 | 135 | 6 | 25 | 99 | 50 | 96 |
| 9, 10 & 11yo | 109 | 1 | 1 | 100 | 7 | 12 | 99 | 50 | 93 |
| All | 480 | 4 | 19 | 438 | 19 | 17 | 96 | 17 | 96 |

## 3. Sensitivity and specificity for strabismus vs amblyopia

There was little evidence suggesting a difference in sensitivity/specificity for strabismus vs amblyopia. Below, we present our analysis for both these conditions, which were pooled into “binocular vision problem” in the main paper.

## Strabismus

We took strabismus to be a detectable tropia on our cover test.

|  | N | TP | FP | TN | FN | Sensitivity | Specificity | PPV | NPV |
| --- | --- | --- | --- | --- | --- | --- | --- | --- | --- |
| 2 & 3yo | 203 | 1 | 17 | 180 | 5 | 17 | 91 | 6 | 97 |
| 4 & 5yo | 207 | 3 | 5 | 198 | 1 | 75 | 98 | 38 | 99 |
| 6 & 7yo | 266 | 2 | 4 | 254 | 6 | 25 | 98 | 33 | 98 |
| 9, 10 & 11yo | 216 | 3 | 4 | 204 | 5 | 38 | 98 | 43 | 98 |
| All | 892 | 9 | 30 | 836 | 17 | 35 | 97 | 23 | 98 |

Table 3. Predictive value of Randot Preschool in predicting those who failed our cover-test, by age-group, taking “pass” as a score of 800 arcsec or lower. N = number of children, TP = number of true positives (those who failed the cover test and failed the stereotest), FP = number of false positives, TN = number of true negatives, FN = number of false negatives, PPV = positive predictive value, NPV = negative predictive value.

## Amblyopia

We took amblyopia to be an interocular acuity difference exceeding 0.2 logMAR, or moderate visual impairment in either eye, or a parental questionnaire suggestive of amblyopia (e.g. child has been treated with drops in an eye clinic). For this analysis, we excluded the nursery children, since visual acuity was not measured in nursery sessions and thus amblyopia would be unlikely to be detected.

|  | N | TP | FP | TN | FN | Sensitivity | Specificity | PPV | NPV |
| --- | --- | --- | --- | --- | --- | --- | --- | --- | --- |
| 4 & 5yo | 207 | 2 | 6 | 198 | 1 | 67 | 97 | 25 | 99 |
| 6 & 7yo | 266 | 3 | 3 | 256 | 4 | 43 | 99 | 50 | 98 |
| 9, 10 & 11yo | 216 | 5 | 2 | 200 | 9 | 36 | 99 | 71 | 96 |
| All | 892 | 10 | 29 | 838 | 15 | 40 | 97 | 26 | 98 |

Table 4. Predictive value of Randot Preschool in predicting amblyopiat, by age-group, taking “pass” as a score of 800 arcsec or lower.
