# Supplementary material for: Characterizing the Randot Preschool stereotest: Testability, norms, reliability, specificity and sensitivity in children aged 2-11 years
Source: PLoS One. 2019 Nov 7;14(11):e0224402. doi: 10.1371/journal.pone.0224402 (PMC6837395; doi:10.1371/journal.pone.0224402)
Supplement: Attachment — Submitted filename: ResponsetoReviewers.docx [file pone.0224402.s002.docx]

*Reviewer #1: The study evaluates the normative values, testability, and reliability of the Randot Preschool stereotest by age and sex in children 3-11. It also assesses sensitivity and specificity of the Randot Preschool to detect binocular vision issues. The strength of the study is to have a very large sample (>1000 children) and precise procedures. The manuscript is very well written, clear, and easy to read. Each result is appropriately backed up with data and statistical tests and conclusions match the data. Each result is discussed in a very interesting way.*

Many thanks for these kind comments, which are much appreciated.

*I have only a few comments, mostly minor.
Minor comments:
pp5 line 93 = « values in italics » - there is no values in italics. Also it is not clear why the ref 13 is in red, given it is not your data, isnt't it?*

Apologies, this formatting was inadvertently mangled while preparing the ms for PLOS One’s submission process. Now fixed – thanks!

*Pp5 – your threshold values are systematically above the other studies. I have seen that you discuss why later. I suspect it comes from the instructions: you used PEDIG instructions while the other studies may have used manufacturer instructions. The manufacturer proposes to start at 200” rather at 800”, and then to go up or down depending on success. It certainly increases the probability to be successful only by chance which lowers the average score. The PEDIG instructions certainly lead to more accurate estimations of scores.*

Many thanks for this very helpful suggestion, which we have added. While we find the suggestion extremely plausible, the data doesn’t seem to bear it out: Afsari et al. began with 800 arcsec as in PEDIG, while Birch et al began with 200 arcsec, yet achieve very similar results. We now discuss this.

*Pp6 – line 124 : “We also excluded any reporting values solely in a clinical population, except that we did include reliability measures in clinical populations of the relevant ages.” I do not understand what you mean here.*

Apologies for being unclear. We have reworded this as follows: “We excluded any studies which contained data solely for ages over 10 years. For normative values, sensitivity and specificity, we also excluded any studies reporting values solely in a clinical population.We did include reliability measures assessed in clinical populations of the relevant ages.” and hope this is now clearer. Our rationale was that we did not necessarily expect a difference in test reliability for children with strabismus etc, whereas of course there would be a difference in normative values. There were no studies of testability purely in a clinical population.

*Pp8 – line 170 and pp12 line 278 – if I understand correctly, around half of the children did not have a questionnaire filled by the time of the screening and therefore were not asked to wear glasses during testing (even if some of them might need glasses). The fact that they are not wearing glasses could explain why their stereoacuity and visual acuity is lower. Do you know what procedure other studies used regarding the correction of refraction when testing stereopsis? Could it explain the larger stereoacuity values that you obtain (if other studies actually systematically measured refraction and used frame glasses for stereopsis testing, or excluded the children with no known prescription)?*

Yes, agreed. We do allude to this in the relevant discussion section, and have expanded on this in response to the reviewer’s comment.

*Pp13 line 293 and 300: the main result here seems to be that testability is virtually 100% after age 4.Given you have many children, would it make sense to do a statistical test (possible showing whether a confidence interval includes 1)? I think this could show that the testability is 1 at least from age x.*

In a strict statistical sense, any failures mean that the true probability cannot be 1. The confidence interval around 95% may include 90%, but cannot include 100%. Of course, this is of little importance. As the reviewer says, the main result is that testability is “virtually 100% in [children older than 3]”, as we state.

*Pp20 – please provide a measure of goodness of fit for your model.*

Now added (legend of Table 7).

*Would you be able to justify whether it is necessary to have 8 parameters rather than 2 or 3, using any procedure to compare models (ex: AIC)? It looks to me that the distributions in Fig 8 could be described by a gamma distribution with two parameters, provided that age and sex can at least influence the scale parameter. You could compare your model to this one.*

This is an interesting suggestion, but we are not really trying to establish which of several models is correct. Our model is purely descriptive, not theory-based, and offers future workers a relatively simple formula for getting the expected distribution of scores (not just means) for a given age and sex. A gamma function model might indeed have fewer parameters, but the disadvantage is that specialised software is needed to evaluate a gamma function. Our model only requires exponentials, which can be done on a calculator.

*Pp23 – line 541 – missing word? “Figure” 10.*

Thank you, quite right!

*Pp23 – line 546– why include condition (c)? It does not seem to be a binocular vision problem.*

Children with poor enough vision in one or both eyes will certainly score badly on a stereotest. We chose 0.48 logMAR, the WHO threshold for “moderate visual impairment”, as our definition of “poor enough”, reasoning that such children will lack the acuity to resolve the dot pattern on a Randot stereotest. (We experimented with other values and did not find anything obviously better).

*Pp24 – line 588- I do not think that specificities can be considered “high” or “excellent”’ in regards to detecting participants with no binocular problem considering that the proportion of children with binocular problems is so low (4% - the target proportion). When the target proportion is that low, it is crucial that specificity is extremely high to avoid drastically decreasing the positive predictive value normalized to the target proportion. For example, if sensitivity is 100% and specificity is 96%, positive predictive value normalized to the target proportion (4%) is only 50%. Could you report positive and negative predictive values normalized to the target population as well?*

Thank you for this helpful suggestion; we have done so (Table 8).

*Abstract: you report in the abstract that 6% of the children are stereoblind but you do not mention it in the text. Do you mean that 6% of all testable children are stereoblind or 6% of children with otherwise normal vision?*

Apologies for being unclear. Due to lack of space in the abstract, we simply pooled across age-groups which are presented individually in the main text. This is for testable children with apparently normal vision. Table 6 gives the results by year-group.

*Reviewer #2: Review of Randot paper*

*This paper presents compressive data on the Random Preschool Stereotest administered to over 1000 children aged 2-11 years. However, its purpose is unclear because there are published data on every one of the measures the authors provide. They state their goal as getting the measures all in the same study but it is not clear why testability from one cohort and scientist and sensitivity on another cohort measured by a different scientist are any less valuable than the data presented here in separate sections of the paper for each measure (and sometimes different cohorts). The authors need to provide a stronger rationale.*

According to the principles of Open Science, even a straight replication has value and merits publication in a journal like PLOS ONE, which explicitly does not require novelty. In fact our paper does contribute new information.

1. Even though results with the Randot Preschool Stereotest have been previously published, results from a different research group in a different country in a different set of children and with slightly different methodology (noted in the text) are useful because, to the extent they are similar, they increase confidence, and to the extent that they differ, they indicate the variability between populations / study methods.

2. Specifically, we extend previous data to include testability/reliability/validity in older children and improved sample sizes for normative data in older children.

3. Previous studies have reported different statistics (e.g. mean/median in arcsec / log-arcsec), making comparisons and meta-analyses difficult. We are the first to publish our full data and analysis code. This will enable future researchers to query our data further, e.g. to obtain percentiles rather than just means and SDs.

4. We find a sex difference which has not previously been reported, perhaps because few studies have been as large as ours.

5. We have summarised our data into formulae giving the probability of obtaining each score as a function of age and sex, which we believe will be of use to future clinicians and researchers.

*The measures are also limited by a ceiling effect (no value can be tested finer than 40 arcsec). That means there is very little variance in the data from children above age 5, severely limiting the possibility of valid measures of reliability or meaningful norms.*

We completely agree. But despite its name, the Randot Preschool stereotest is sometimes used clinically in children over 5, so it's important to quantify its performance in these groups.

*For the tests of sensitivity and specificity, the outcome will depend on the cutoffs that are used, which likely need to differ by age up to age 6—as is clear in Figure 10. This makes Figure 9 meaningless.*

(should this be Figure 10?) We disagree that Figure 10 (or indeed 9!) is meaningless. It is divided into subpanels by age-group, so if it were the case that clear cut-offs existed for each age-group, we would be able to see this. In fact, the problem is that there is no clear cut-off at age-group. In each case, there are some children who clearly have age-normal stereoacuity yet failed the cover test, and others who score nil yet have no detectable abnormalities. We now discuss this in the text.

*The small number of children with binocular problems on other measures and the variety of other measures used also make the sensitivity data difficult to interpret. A brief report on the measures that are not limited by the ceiling—i.e., for the younger children only—could be of interest if the authors explain how it differs from the existing literature.*

We agree that the rare prevalence of binocular problems is a drawback, but still consider our data worth reporting, especially since we are aware of only two earlier studies (refs 12,13). In fact, as shown in Table 1, our results are in line with earlier studies.

*The sex difference is interesting and reminiscent of the one identified during infancy on the onset of stereopsis, although that sex difference may be an consequence of sex differences in accommodative and vergence eye movements:*
*Gwiazda, J., Bauer, J., & Held, R. (1989). Binocular function in human infants: correlation of stereoptic and fusion-rivalry discriminations. J Pediatr Ophthalmol Strabismus, 26(3), 128-132. Retrieved from* [*https://www.ncbi.nlm.nih.gov/pubmed/2723974*](https://www.ncbi.nlm.nih.gov/pubmed/2723974) *Horwood, A. M., & Riddell, P. M. (2008). Gender differences in early accommodation and vergence development. Ophthalmic Physiol Opt, 28(2), 115-126. doi:10.1111/j.1475-1313.2008.00547.x*

We thank the reviewer very much for these helpful references, now added.

*Minor points:
1, The authors transform all values to log units because they are more likely to be normally distributed. That’s a sound rationale but makes it very difficult to relate the findings to the actual 6 value it is possible to get on the test.*

To avoid this problem, we never give a result in log units without translating it to the more familiar units. Thus, any single value in log arcsec is immediately followed by the corresponding value in arcsec, while any range in log arcsec is immediately followed by its value as a factor (e.g. a range of 0.3 log arcsec corresponds to a factor of 2). The only exception we could find was in the Reliability Discussion section, where having given our results (“0.64 log arcsec or a factor of 4.3”) we then compared them with previous results given in log arcsec only. We have now added these and would be grateful to know of any further omissions.

*2, The acuity chart is inappropriate for children older than 4 because there are no values smaller than 0.2 loMAR. Children of age 5+ usually achieve an acuity of logMAR .10. This limits the usefulness of the analysis of sensitivity and specificity.*

As explained in the Methods, the limit of 0.2 logMAR applied only to 4 and 5 year old children participating in the Orthoptic Screening Programme. For children older than 5, we tested to threshold. This is apparent in Fig 2, where you can see children older than 5 are scoring on average around logMAR 0.10, as the reviewer says, with many children scoring even better.

*3, The interocular acuity difference consider indicative of abnormal binocular vision was greater than logMAR .20 (which is more than 2 lines). Amblyopia is normally defined as a difference of 2 lines or more.*

We had chosen the value of 0.3 since it resulted in slightly higher sensitivity, but can see the merit of using the accepted value, and have modified our results accordingly.

*4, Taking 800 arcsecs (Table 8) is an inappropriate cutoff because by age 4 almost all children score better than that.*

That’s actually a reason in favour of 800 arcsec - one would wish to choose a cut-off for which almost all visually normal children score better than that, leaving only children with binocular vision problems scoring worse. We did consider different cut-offs, but as you can see from Figure 10, there is no cut-off which clearly separates children with binocular vision problems from those without.

*5, Because there are only 6 discrete values and the test is not designed to determine a threshold, it’s not surprising that reliability is poor. The authors should make this point and not spend so much space analyzing the reliability data.*

 Actually, these features of the test should improve apparent reliability. Consider a test which scores patients to the nearest arcsec. A patient might score 320 arcsec on his first test and 590 on re-test, resulting in a discrepancy of 270 arcsec or 60% of the mean score. On Randot Preschool, where only scores of …200,400,800 are available, this person would score 400 arcsec both times, i.e. perfect agreement. We do make this point in the paper (Reliability Discussion section).

We think it’s important to quantify reliability, particularly as we reveal an effect of age on reliability which has been missed (though was present in the data) in earlier studies.
