# Supplementary material for: Characterizing the Randot Preschool stereotest: Testability, norms, reliability, specificity and sensitivity in children aged 2-11 years
Source: PLoS One. 2019 Nov 7;14(11):e0224402. doi: 10.1371/journal.pone.0224402 (PMC6837395; doi:10.1371/journal.pone.0224402)
Supplement: Attachment — Submitted filename: RevComms.docx [file pone.0224402.s003.docx]

Additional Editor Comments (if provided):

Minor Comments:

1) I believe the x axis in figures 8 and 10 should read “Randot stereoacuity (arcsec)” instead of “Age-group”.

*Apologies, yes that would be clearer. Change made.*

2) I know you have correctly uploaded your data to a public repository, but I’ve also noted that in a few places you reference analyses without reporting them. The PLOS guidelines state:

“Please note that PLOS does not permit references to “data not shown.” Authors should provide the relevant data within the manuscript, the Supporting Information files, or in a public repository. If the data are not a core part of the research study being presented, we ask that authors remove any references to these data.”

Therefore, I ask you to add these analyses as brief supporting information. Specifically, I am referring to:

Page 22 line 515: “data not shown”
Page 24 line 580: “not shown”
Page 25 Line 615 “Some authors have suggested that random-dot stereotests are more sensitive to strabismus than to amblyopia, but neither our data nor that of Afsari et al(13) suggests a significant difference in sensitivity.”

*As you note, these analyses were already performed in the R markdown files provided in the repository, but I now also include them in a separate Supporting Information document.*

3) On page 24 line 585 you say that crosstalk “makes it possible to pass the higher levels of the test with one eye”. I think you should explain briefly how this might work, as it is not very intuitive. Additionally, you should mention that crosstalk might also worsen your results, since crosstalk decreases perceived depth:
Tsirlin, I., Wilcox, L. M., & Allison, R. S. (2012). Effect of crosstalk on depth magnitude in thin structures. Journal of Electronic Imaging, 21(1), 011003.

*I have added text and a figure to explain this.*

[Note: HTML markup is below. Please do not edit.]

Reviewers' comments:

Reviewer's Responses to Questions

**Comments to the Author**

1. If the authors have adequately addressed your comments raised in a previous round of review and you feel that this manuscript is now acceptable for publication, you may indicate that here to bypass the “Comments to the Author” section, enter your conflict of interest statement in the “Confidential to Editor” section, and submit your "Accept" recommendation.

Reviewer #1: All comments have been addressed

*Thank you, I am happy to hear this!*
